# Effects of D-Tagatose on Cariogenic Risk: A Systematic Review of Randomized Clinical Trials

**DOI:** 10.3390/nu17020293

**Published:** 2025-01-15

**Authors:** Lissé Angarita-Davila, Héctor Fuentes-Barría, Diana Rojas-Gómez, Raúl Aguilera-Eguía, Miguel Alarcón-Rivera, Eduardo Guzmán-Muñoz

**Affiliations:** 1 Escuela de Nutrición y Dietética, Facultad de Medicina, Universidad Andres Bello, Concepción 3349001, Chile; lisse.angarita@unab.cl; 2 Vicerrectoría de Investigación e Innovación, Universidad Arturo Prat, Iquique 1100000, Chile; 3 Escuela de Ondontología, Facultad de Odontología, Universidad Andres Bello, Concepción 3349001, Chile; 4 Escuela de Nutrición y Dietética, Facultad de Medicina, Universidad Andres Bello, Santiago 7550000, Chile; diana.rojas@unab.cl; 5 Departamento de Salud Pública, Facultad de Medicina, Universidad Católica de la Santísima Concepción, Concepción 3349001, Chile; raguilerae@ucsc.cl; 6 Escuela de Ciencias del Deporte y Actividad Física, Facultad de Salud, Universidad Santo Tomás, Talca 3460000, Chile; mrivera3@santotomas.cl; 7 Facultad de Medicina, Universidad Católica del Maule, Talca 3460000, Chile; 8 Escuela de Kinesiología, Facultad de Salud, Universidad Santo Tomás, Talca 3460000, Chile; eguzmanm@santotomas.cl; 9 Escuela de Kinesiología, Facultad de Ciencias de la Salud, Universidad Autónoma de Chile, Talca 3460000, Chile

**Keywords:** tagatose, dental caries, *Streptococcus mutans*, biofilms

## Abstract

Dental caries remains a prevalent chronic disease driven by dysbiosis in the oral biofilm, with *Streptococcus mutans* playing a central role in its pathogenesis. Objective: This study aimed to assess the effect of D-tagatose on cariogenic risk by analyzing randomized clinical trials (RCTs). Methods: A systematic literature review was conducted targeting RCTs published up to 2024 in eight databases and two gray literature sources. The search strategy utilized Medical Subject Headings (MeSHs) and relevant keywords combined via Boolean operators using the query “Tagatose OR D-tagatose AND Dental Caries”. Eligible studies must evaluate the impact of D-tagatose on cariogenic risk, as indicated by reductions in colony-forming units (CFUs) and/or improvements in salivary pH levels in treatment groups. Results: From 1139 retrieved records, three studies met the inclusion criteria. Two of these studies consistently demonstrated significant reductions in CFU counts in vitro and changes in oral bacteria in groups treated with D-tagatose alone or in mixtures with other agents compared to controls using other non-caloric sweeteners or placebos (*p* < 0.01). However, the quality of the evidence was heterogeneous, with certain methodological concerns. Conclusions: Although the findings suggest potential benefits of D-tagatose in reducing cariogenic risk, limitations such as small sample sizes and variability in study methodologies warrant caution. Further robust investigations are needed to substantiate these promising results and support the integration of D-tagatose into oral care formulations aimed at reducing cariogenic risk.

## 1. Introduction

Dental caries is among the most prevalent non-communicable chronic diseases globally, affecting individuals across all age groups. It typically begins after tooth eruption and progresses through the demineralization of dental hard tissues, ultimately leading to cavity formation [1,2,3]. These cavities significantly impair oral health and quality of life. If left untreated, dental caries becomes a leading cause of oral pain, severe infections, and tooth loss, with profound effects on masticatory function and psychological well-being [4,5,6].

The etiology of dental caries is closely linked to dysbiosis within the oral biofilm—a dynamic, structured microbial community that adheres to dental surfaces. This biofilm comprises bacteria, fungi, and other microorganisms and is maintained through interactions among microbial species, saliva, and tooth surfaces [7,8]. A central factor in this imbalance is the activity of glucosyltransferase (GTF) enzymes, which play a pivotal role in the pathogenesis of dental caries. GTFs, which are primarily expressed by cariogenic bacteria such as *Streptococcus mutans*, catalyze the synthesis of glucans from dietary sugars, particularly glucose [7,8].

These glucans, consisting of both soluble and insoluble glucose polymers, form a matrix that enhances bacterial adhesion to tooth enamel and strengthens the structural integrity of the biofilm. This matrix not only increases bacterial resistance to mechanical removal but also provides protection from host defense mechanisms, including leukocytes and salivary antimicrobial agents [6,9]. Additionally, insoluble glucans trap nutrients and sugars within the biofilm, creating an environment conducive to bacterial proliferation. During sugar metabolism, bacteria produce organic acids, such as lactic acid, which lower the pH in the oral cavity. This acidic environment promotes enamel demineralization, accelerating the development and progression of carious lesions [1,10,11].

Within the biofilm, *Streptococcus mutans* utilizes sugars as an energy source, enhancing bacterial adhesion to tooth surfaces while generating acidic byproducts. These byproducts further reduce the pH of the oral environment, exacerbating enamel demineralization and facilitating caries progression. This multifaceted disease process highlights the interplay of bacterial, enzymatic, and environmental factors underlying the progression of dental caries [1].

In this context, D-tagatose has emerged as a novel sweetener with significant antimicrobial properties, offering promising potential for caries prevention [1]. This rare sugar, characterized by a caloric value markedly lower than that of sucrose, uniquely inhibits GTF activity, thereby reducing glucan production and bacterial biofilm formation. Remarkably, D-tagatose retains its efficacy even in the presence of sucrose, a primary cariogenic sugar [1,12,13]. Beyond limiting bacterial adhesion, D-tagatose modulates the oral environment, providing a comprehensive approach to caries prevention (as summarized in Table 1).

Recent research suggests that D-tagatose surpasses other sweeteners, such as xylitol, in mitigating cariogenic processes. Unlike xylitol, D-tagatose maintains its acid-neutralizing capacity even when combined with sucrose, providing a distinct advantage in reducing caries risk. These antimicrobial properties, coupled with its ability to regulate the oral environment, position D-tagatose as a promising agent for preventing dental caries and addressing biofilm-related imbalances [10]. Consequently, D-tagatose represents a cornerstone of preventive strategies targeting caries and other oral disorders linked to biofilm dysbiosis [7,8,9,11,13].

Considering these findings, this study aimed to systematically evaluate the effects of D-tagatose on the oral environment, with a specific focus on its influence on microbial activity and biofilm formation. Furthermore, this study sought to explore the potential of D-tagatose as a preventive agent for mitigating caries development, examining its mechanisms of action, effectiveness in reducing bacterial adherence, inhibition of glucosyltransferase activity, and modulation of oral cavity pH levels.

## 2. Materials and Methods

### 2.1. Design

The protocol adhered to the guidelines of the Preferred Reporting Items for Systematic Reviews and Meta-Analyses (PRISMA); approval code: INPLASY202510002 and approval code: PROSPERO CRD42025632489. It was developed following the recommendations outlined in the Cochrane Handbook for Systematic Reviews of Interventions [14,15].

### 2.2. Eligibility Criteria

Eligibility for study inclusion was determined using the P.I.C.O.S framework, which includes participants, intervention, comparison, outcome, and study design, with the following specifications:

- Participants: adults with oral health characterized by the absence of active caries; advanced periodontitis; and significant oral lesions associated with smoking, excessive alcohol consumption, or diets extremely high in simple sugars.
- Intervention: administration of D-tagatose as a standalone intervention.
- Comparison: the use of any non-caloric sweetener other than D-tagatose (e.g., sucrose, stevia, or xylitol).
- Outcome: changes in colony-forming units (CFUs) or salivary pH.
- Study design: randomized controlled trials (RCTs) with variable intervention durations.

RCTs were prioritized due to their ability to provide more reliable results with a lower risk of bias. Non-randomized studies were excluded to minimize bias and ensure accurate meta-analysis results.

### 2.3. Data Sources and Search

A comprehensive electronic search was performed in December 2023 and updated in December 2024. The databases consulted included Web of Science (accessed on 31 December 2024), Europe PMC (accessed on 31 December 2024), Scopus (accessed on 31 December 2024), Medline/PubMed (accessed on 31 December 2024), CENTRAL (accessed on 31 December 2024), Dentistry & Oral Sciences Source (accessed on 31 December 2024), Springer Link (accessed on 31 December 2024), and the Virtual Health Library (accessed on 31 December 2024). Gray literature was also included, with searches conducted in bioRxiv (accessed on 31 December 2024) and at https://www.preprints.org/ (accessed on 31 December 2024).

The search strategy utilized Medical Subject Headings (MeSHs) terms combined with Boolean operators (AND/OR) to construct the following query across all databases: “Tagatose OR D-tagatose AND Dental Caries”. The search results were not limited in their language and were subsequently filtered by methodological design, specifying “Article” in Scopus and Web of Science, “Research Articles” in Europe PMC and Springer Link, and “Randomized Controlled Trial” in Medline/PubMed (Table S1 in the Supplementary Material).

### 2.4. Study Selection and Data Collection

Two researchers (L.A.D. and H.F.B.) independently screened titles, abstracts, and full texts for eligibility. Discrepancies were resolved by a third reviewer (R.A.E.), who acted as an arbitrator. Key data, including the primary author, publication year, study population, comparisons, main outcomes, and methodology, were extracted. Missing or unclear data prompted direct communication with the corresponding author.

### 2.5. Risk of Bias

The methodological quality of the included studies was evaluated using the Risk of Bias 2 (ROB 2) tool developed by the Cochrane Collaboration. This tool assesses and classifies potential biases in randomized controlled trials across five key domains: (D1) the random sequence generation process, (D2) deviations from assigned interventions, (D3) missing outcome data, (D4) outcome measurement, and (D5) selection of reported outcomes [16].

Subsequently, the Grading of Recommendations Assessment, Development, and Evaluation (GRADE) approach was applied to assess the quality of evidence and the strength of recommendations by evaluating the certainty of the results. Primary outcomes were identified, and key aspects such as the study methodology, risk of bias, consistency, precision, and applicability were assessed. Each outcome was classified into one of four categories: high, moderate, low, or very low quality. The evidence quality was adjusted based on the effect size, confidence intervals, and confounding factors. Recommendations were made considering the results, evidence quality, and patient preferences [17].

The risk of bias and quality of evidence were assessed independently by two researchers (H.F.B. and R.A.E.). Discrepancies regarding the inclusion of specific articles were resolved by a third reviewer (E.G.M.), who acted as an arbitrator. This structured process allowed for clear and transparent conclusions regarding the quality of the evidence in the review.

### 2.6. Strategy for Data Synthesis

The extracted data were used for a descriptive summary analysis, adhering to systematic review guidelines. Homogeneous variables underwent meta-analysis using Stata 17. Heterogeneity was assessed with the *I*^2^ test and categorized as substantial (>75%), moderate (40–75%), or low (<40%). A random-effects model was applied to analyze the data.

## 3. Results

**Figure 1:**
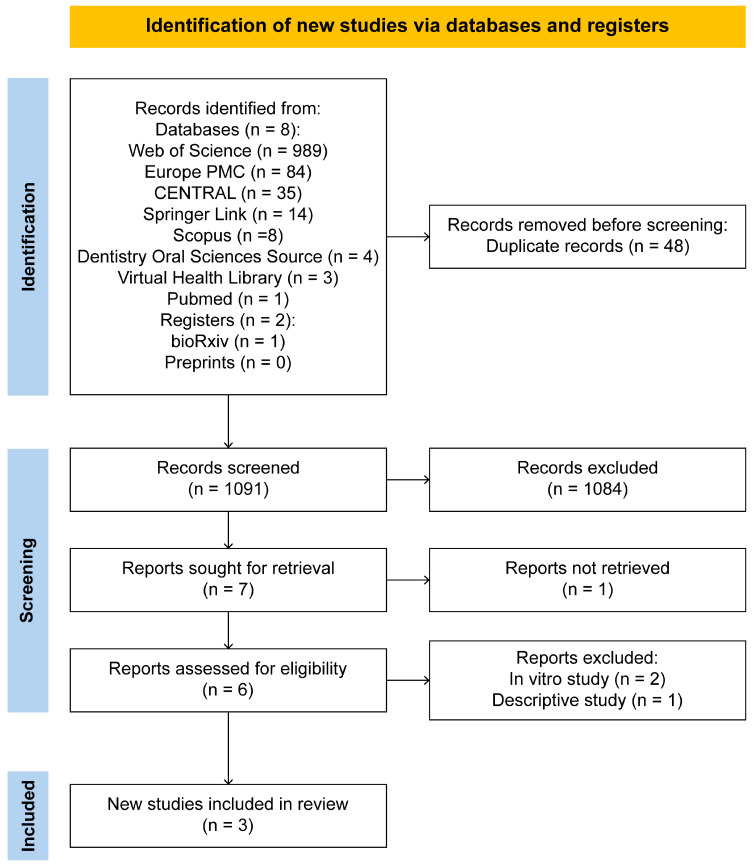
The studies included in the systematic review.

Figure 1 illustrates the search process conducted on 31 December 2024. A total of 1138 records were identified across the databases, along with 1 record from the gray literature (the bioRxiv website; no records were retrieved from preprints.org) [18].

All records were manually screened by two independent authors, with a third reviewer serving as an arbitrator to resolve disagreements.

This initial process led to the identification of 48 duplicate records. The remaining 1091 records were evaluated based on their titles, abstracts, and keywords, resulting in the exclusion of 1084 records that did not meet the relevance criteria for the study topic. The seven remaining articles were selected for full-text eligibility assessment [7,8,10,13,19,20,21]. Of these, one article was excluded, as the full text could not be retrieved [21]. Subsequently, from the six fully recovered and reviewed articles, two were excluded for using in vitro models [19,20] and one for its descriptive observational design [8]. Finally, three articles were included in the analysis of this review [7,10,13] (Table S2 in the Supplementary Material).

Table 2 summarizes the main characteristics of the studies included in the analysis.

The study by Nagamine et al. [7], conducted in Japan, included 19 healthy volunteers (ages: 21–49 years) and focused on reductions in *Streptococcus mutans* CFU/mL utilizing quantitative PCR and microbiological cultures.

The study by Urrutia-Espinosa et al. [10], conducted in Chile, included 30 students (ages: 18–30 years) and evaluated the effects of different sweeteners on salivary pH and total CFU/mL using a calibrated digital pH meter and microbiological cultures.

The study by Zakis et al. [13], conducted in the Netherlands, involved 65 adults (ages 18–55 years) and assessed the effect of tagatose on salivary pH and the oral microbiome using 16S rRNA gene amplicon sequencing, red fluorescence of plaque, and a calibrated pH meter.

Table 3 presents the main characteristics of the intervention programs used in the studies by Nagamine et al. [7], Urrutia-Espinosa et al. [10], and Zakis et al. [13].

In the study by Urrutia-Espinosa et al. [10], the participants received mouth rinses containing D-tagatose (EG1), stevia (EG2), and sucrose (CG) at concentrations of 6.4% for 48 h.

The study by Nagamine et al. [7] implemented a 4-week intervention with chewing gums containing 5% xylitol (EG1), 5% D-tagatose (EG2), a combination of 2.5% D-tagatose and 2.5% xylitol (EG3), and a placebo (CG).

In the study by Zakis et al. [13], the participants received mouth rinses D-tagatose, Glucose, Inulin, Isomaltulose and Trehalose at concentrations of 10% for 2 weeks with final observations at the end of 4 weeks.

Table 4 summarizes the key findings from the interventions with alternative sweeteners.

The study by Nagamine et al. [7] reported significant decreases in total CFU/mL and the *Streptococcus mutans* level in the experimental group receiving D-tagatose + xylitol compared to the control group.

In the study by Urrutia-Espinosa et al. [10], significant reductions in colony-forming units (CFU/mL) were observed in the experimental groups (G1 (stevia) and G2 (D-tagatose)) compared to the control group (CG (sucrose)).

No significant differences were identified between the experimental groups (G1 vs. G2).

The study by Zakis et al. [13] found that mouth rinses with 10% D-tagatose did not significantly change the oral microbiome compared to other sweetener groups. Additionally, no significant changes in salivary pH were observed.

**Figure 2:**
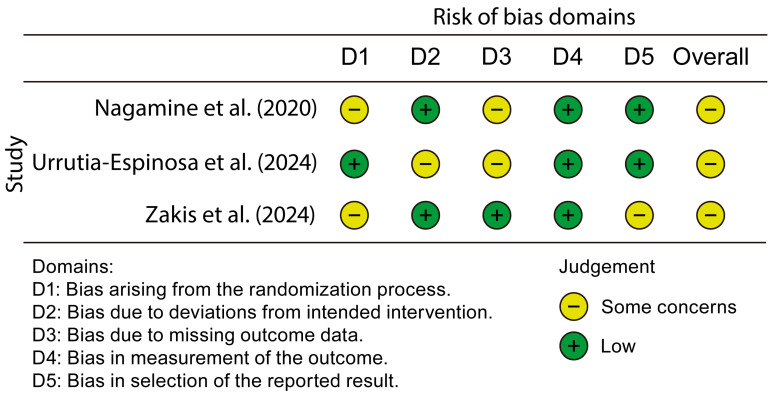
The risk of bias in the studies included in the systematic review. Source: Nagamine et al. [7], Urrutia-Espinosa et al. [10], Zakis et al. [13].

Figure 2 summarizes the findings of the risk-of-bias assessment performed using the ROB 2 tool for the selected studies, highlighting both strengths and limitations based on the evaluated domains.

In the case of Nagamine et al. [7], some concerns were identified regarding randomization. While a double-blind design was mentioned, critical details, such as how the random sequence was generated or assigned, are lacking, which introduces uncertainty. As for blinding, the double-blind design was executed appropriately, with no evidence of significant deviations in the interventions applied. However, it was noted that the measurements were taken by a single investigator, and it was not confirmed whether the investigator was blinded, leaving room for potential bias. Additionally, although two participants were excluded for justified reasons, these exclusions did not affect the overall results. The reporting of the results was thorough, with appropriate statistical analyses and no evidence of selective reporting.

In contrast, the study by Urrutia-Espinosa et al. [10] presented a low risk of randomization, as an online random sequence generator was used, along with stratified allocation by the center, thereby ensuring proper randomization. However, the blinding method was simple, which raises concerns about potential staff influence, though no clear evidence of deviations was found. Regarding the measurements, although calibrated tools were used to assess pH and CFUs, it was not specified whether the evaluators were blinded, which may have introduced bias. Data handling was appropriate, with no participant losses, and all data were included in the analysis, ensuring integrity. Additionally, all predefined outcomes were reported in line with the study objectives, and the CONSORT guidelines were followed.

Finally, the study by Zakis et al. [13] showed strong implementation of the double-blind design, with rigorous monitoring of compliance through the return of used vials by the participants. Automated methods, such as genomic sequencing, were employed to minimize subjective influence in measuring outcomes, reducing bias. Furthermore, the dropout rate was low (6%), and missing data were appropriately handled through intention-to-treat analysis. However, while key results were reported, it was not clear whether all predefined outcomes were included or whether selective reporting occurred. In the study by Zakis et al. [13], Figures 3–5 offer only a visual summary with limited statistical depth, based solely on *p*-values and frequency percentages, without complementary inferential metrics such as confidence intervals or effect sizes.

Although included in the tables provided, they include different results and do not clarify or complement the data shown in the figures, potentially obscuring the full scope and interpretation of the results.

Overall, all three studies demonstrate solid methodologies, but each presents areas for improvement, particularly regarding randomization, the blinding of measurements, and the reporting of results. These uncertainties are crucial when evaluating the validity and reliability of the studies (Table S3 in the Supplementary Material).

The data presented in Table 5 show the results of the meta-analysis of CFUs and salivary pH at different time intervals, comparing D-tagatose with stevia and sucrose. They show that the evidence quality is very low (GRADE level).

Only the study by Urrutia-Espinosa et al. [10] could be properly meta-analyzed, which may limit the interpretation and generalization of the results. Overall, small mean differences and wide confidence intervals are observed, reflecting uncertainty related to the effects of D-tagatose compared to stevia and sucrose for both measured parameters (Figure S1 in the Supplementary Material).

## 4. Discussion

The results of this systematic review highlight the potential of D-tagatose as an effective agent in preventing dental caries, particularly by inhibiting the proliferation of cariogenic bacteria such as *Streptococcus mutans*. These findings emphasize the importance of exploring alternatives to mitigate the adverse effects of sugar-rich diets, a significant factor contributing to the global prevalence of dental caries [7,9,10].

When D-tagatose has been incorporated into oral care products, especially when combined with xylitol, it has demonstrated notable antimicrobial activity against *Streptococcus mutans* and other bacteria present in human saliva. This compound inhibits bacterial growth and the production of insoluble glucans, essential components of biofilm formation, even in the presence of sucrose [11,19,20]. Bacterial biofilms, which shield bacteria from host defenses and antimicrobial treatments, represent a critical target in caries prevention [22,23,24,25,26,27].

Studies have shown the efficacy of combining D-tagatose and xylitol, as evidenced in chewing gum formulations, where their combined effects surpass the benefits of either compound used alone [7,24,28,29]. Additionally, D-tagatose, when used as a mouth rinse, has demonstrated superior performance compared to sucrose and is comparable to stevia in the short term [10].

In the context of modern sugar-rich diets, D-tagatose distinguishes itself by disrupting the metabolic activity of *S. mutans* and other oral bacteria. It inhibits glycolysis and reduces the expression of glucosyltransferases (GTFs), such as GTF-B, thereby limiting the production of glucans essential for bacterial adherence and biofilm development [11,20,30,31,32]. Furthermore, its low glycemic index and prebiotic properties support the growth of beneficial microorganisms in the colon, aided by fermentation by resident bacteria [33,34,35,36].

Moreover, D-tagatose stimulates glycogen synthesis, lowering plasma glucose levels and, consequently, reducing the substrate available for cariogenic bacteria within the oral biofilm [12,13,37]. These attributes, alongside its antioxidant capacity to eliminate free radicals, contribute to reducing oxidative stress at the cellular level. Its positive influence on glycolysis, glycemic control, and insulin responses positions D-tagatose as a promising alternative for managing diabetes mellitus and obesity. Furthermore, its application in products like toothpaste, chewing gum, and functional foods enhances its relevance in caries prevention [7,10,11,19,37].

One of the key strengths of this review is the robust evidence supporting the significant antimicrobial potential of D-tagatose, particularly in combination with xylitol [7]. This pairing has demonstrated superior efficacy in reducing bacterial biofilm formation and inhibiting the production of insoluble glucans, which are critical for bacterial adherence [11,19,20]. Additionally, studies indicate that D-tagatose used in mouth rinses achieves outcomes comparable to stevia and exceeds those of sucrose in reducing bacterial activity in the short term [10].

Beyond its antimicrobial effects, D-tagatose provides additional health benefits. Its low glycemic index and prebiotic effects support the growth of beneficial gut microbiota while also contributing to reduced plasma glucose levels [11,12,37]. These characteristics make D-tagatose particularly suitable for individuals with systemic conditions such as diabetes or obesity, where metabolic regulation is essential [38]. Additionally, its antioxidant properties help mitigate oxidative stress, enhancing its potential as a multifunctional agent in oral care [8,11,13,19].

The broad applicability of D-tagatose in oral care products—including toothpaste, chewing gum, and functional foods—further underscores its importance. Its synergistic potential with agents like xylitol, chlorhexidine, and fluoride presents opportunities to develop advanced oral care solutions tailored to high-risk populations [7,20,39].

While D-tagatose demonstrates considerable promise, it is essential to recognize that other alternative sweeteners, such as stevia and xylitol, also offer similar benefits [7,20,40]. Stevia is particularly valued for its natural origin and low caloric content, making it an attractive option for those seeking non-synthetic alternatives [10]. Xylitol, which has been extensively studied due to its antimicrobial and anti-cariogenic properties, has shown significant efficacy in reducing the risk of dental caries [7,11]. Exploring combinations of these sweeteners with D-tagatose could provide valuable insights for creating multifaceted oral care formulations [7,11].

Despite these strengths, this review identified several limitations. The quality of the evidence may have been influenced by the clinical judgments of the evaluators and the limited number of studies retrieved, which may have excluded the gray literature. Additionally, the predominance of in vitro studies restricts the generalizability of the findings to clinical contexts [7,10,13].

The quality of the included evidence could be limited by inconsistencies in methodological reporting, such as a lack of information on blinding, randomization processes, and allocation concealment [41,42]. The limited number of included studies may have reduced the comprehensiveness of the findings, and the significant reliance on in vitro studies limits the translation of the results to clinical contexts [19,20].

Another limitation is the heterogeneity of the study designs, intervention protocols, and outcome measures, which complicates data synthesis [43]. Variability in the D-tagatose concentrations, modes of administration, and study durations further limit the derivation of standardized recommendations [11]. Moreover, most studies focused on short-term effects, leaving gaps in understanding its long-term safety and efficacy, including potential adverse effects and sustained antimicrobial activity [7,10,13].

To advance this field, future studies should prioritize well-designed randomized controlled trials involving diverse populations to validate in vitro findings and standardize protocols for D-tagatose administration, including consistent concentrations and durations. Long-term research into its effects on oral and systemic health, such as its impact on oral microbiota and metabolic parameters, will also be essential [8,10,11].

In conclusion, incorporating D-tagatose into products like toothpaste, mouthwash, and chewing gum offers a promising preventive strategy against dental caries. Its combination with xylitol appears to be particularly effective in high-risk populations, addressing the adverse effects of sugar-rich diets and positioning D-tagatose as an innovative and practical alternative in oral care [7,10].

## 5. Conclusions

In conclusion, the results of this review underscore the promising potential of D-tagatose as an effective agent for inhibiting bacterial proliferation associated with dental caries formation, demonstrating results comparable to xylitol and stevia. However, further clinical and longitudinal studies will be essential to validate these effects and establish the efficacy of D-tagatose in interventions aimed at preventing caries and other oral diseases related to biofilm formation. Additionally, investigating its synergy with antiseptic and remineralizing agents will be crucial in optimizing its therapeutic potential.

## Figures and Tables

**Table 1:** Antimicrobial and beneficial properties of D-tagatose.

Property	Description
Antimicrobial property	Inhibits the growth of pathogenic bacteria in the oral cavity, particularly *Streptococcus* spp.
Plaque reduction	Reduces bacterial biofilm formation on teeth, potentially reducing plaque accumulation.
Effect on oral health	Prevents dental caries and periodontal diseases by inhibiting harmful bacteria.
Low glycemic index	Does not significantly increase blood glucose levels, making it suitable for diabetics.
Antioxidant effect	May reduce oxidative stress and inflammation in oral tissues.
Effect on metabolism	Aids in weight management by preventing fat accumulation.

Sources: adapted from studies by Nagamine et al. [7], Mayumi et al. [8], Urrutia-Espinosa et al. [10], and Ortiz et al. [11].

**Table 2:** The characteristics of the selected studies.

Characteristics	Nagamine et al. [7]	Urrutia-Espinosa et al. [10]	Zakis et al. [13]
Country	Japan	Chile	Netherlands
Sample	19 healthy volunteers (21–49 years)	30 students (18–30 years)	65 healthy volunteers (18–55 years)
Design	RCT	RCT	RCT
Outcome	CFU/mL	CFU/mL Salivary pH	Beta diversity of oral biofilm bacterial taxa Salivary pH.
Instruments	Microbiological crop. Polymerase chain reaction	Microbiological crop. Calibrated pH meter	16S rRNA gene amplicon sequencing, red fluorescence of plaque, and a calibrated pH meter.

RCT: Randomized controlled trial, CFU/mL: colony-forming units/mL. Source: Nagamine et al. [7], Urrutia-Espinosa et al. [10], Zakis et al. [13].

**Table 3:** The characteristics of the intervention programs.

Characteristics	Nagamine et al. [7]	Urrutia-Espinosa et al. [10]	Zakis et al. [13]
Intervention	EG1: chewing gum with xylitol (5%). EG2: chewing gum with D-tagatose (5%). EG3: D-tagatose gum (2.5%) + 2.5% xylitol (2.5%). CG: placebo.	EG1: D-tagatose mouthwashes (6.4%). EG2: stevia mouthwashes (6.4%). CG: sucrose mouthwashes (6.4%).	EG1: D-tagatose mouthwashes (10%). EG2: Glucose mouthwashes (10%). EG3: Inulin mouthwashes (10%). EG4: Isomaltulose mouthwashes (10%). EG5: Trehalose mouthwashes (10%).
Duration of Study	4 wk	48 h	4 wk

EG: experimental group, CG: control group. Source: Nagamine et al. [7], Urrutia-Espinosa et al. [10], Zakis et al. [13].

**Table 4:** Comparison of effects of D-tagatose on CFU/mL across studies.

	Nagamine et al. [7]	Urrutia-Espinosa et al. [10]	Zakis et al. [13]
Results	D-tagatose + xylitol significantly decreased CFU/mL compared to control group (*p* < 0.01).	D-tagatose significantly decreased CFU/mL in comparison to sucrose (*p* < 0.001). D-Tagatose did not show significant changes in CFU/mL compared to stevia (*p* = 0.137).	D-tagatose shows no difference significant on salivary pH or microbiome composition compared to baseline.

CFU/mL: colony-forming units/mL. Source: Nagamine et al. [7], Urrutia-Espinosa et al. [10], Zakis et al. [13].

**Table 5:** The results of the meta-analysis of CFUs and salivary pH at different time intervals.

Outcome	Comparison	Time	MD	95% CI	Evidence Level (GRADE)
CFU/mL	D-tagatose vs. stevia	30 min after	22.60	−16.99 to 62.19	Very low
CFU/mL	D-tagatose vs. sucrose	30 min after	−204.6	−237.85 to −171.35	Very low
Salivary pH	D-tagatose vs. sucrose	30 min after	−0.08	−0.47 to 0.31	Very low
Salivary pH	D-tagatose vs. stevia	30 min after	−0.06	−0.31 to 0.19	Very low
Salivary pH	D-tagatose vs. sucrose	48 h after	0.68	0.19 to 1.17	Very low
Salivary PH	D-tagatose vs. stevia	48 h after	0.23	−0.03 to 0.49	Very low

CFU/mL: colony-forming units/mL, pH: potential of hydrogen, MD: mean deviation, CI: confidence interval. Source: Urrutia-Espinosa et al. [10].

## Data Availability

The data related to this study are available in this article.

## Supplementary Materials

The following supporting information can be downloaded at https://www.mdpi.com/article/10.3390/nu17020293/s1. Table S1. Summary of search strategies; Table S2. Summary of studies evaluated in full text excluded; Table S3. Summary of risk of bias; Figure S1. Meta-analysis of the included studies.
