# Correction: Angarita-Davila et al. Effects of D-Tagatose on Cariogenic Risk: A Systematic Review of Randomized Clinical Trials. *Nutrients* 2025, *17*, 293

**DOI:** 10.3390/nu17172807

**Published:** 2025-08-29

**Authors:** Lissé Angarita-Davila, Héctor Fuentes-Barría, Diana Rojas-Gómez, Raúl Aguilera-Eguía, Miguel Alarcón-Rivera, Eduardo Guzmán-Muñoz

**Affiliations:** 1 Escuela de Nutrición y Dietética, Facultad de Medicina, Universidad Andres Bello, Concepción 3349001, Chile; 2 Vicerrectoría de Investigación e Innovación, Universidad Arturo Prat, Iquique 1100000, Chile; 3 Escuela de Ondontología, Facultad de Odontología, Universidad Andres Bello, Concepción 3349001, Chile; 4 Escuela de Nutrición y Dietética, Facultad de Medicina, Universidad Andres Bello, Santiago 7550000, Chile; 5 Departamento de Salud Pública, Facultad de Medicina, Universidad Católica de la Santísima Concepción, Concepción 3349001, Chile; 6 Escuela de Ciencias del Deporte y Actividad Física, Facultad de Salud, Universidad Santo Tomás, Talca 3460000, Chile; 7 Facultad de Medicina, Universidad Católica del Maule, Talca 3460000, Chile; 8 Escuela de Kinesiología, Facultad de Salud, Universidad Santo Tomás, Talca 3460000, Chile; 9 Escuela de Kinesiología, Facultad de Ciencias de la Salud, Universidad Autónoma de Chile, Talca 3460000, Chile

## Text Correction

There was an error in the original publication [1], regarding a typographical error. A correction has been made to the Abstract in the Methods subsection. The corrected text is as follows:

“and/or”.

There was an error in the original publication [1], as the content is incomplete. A correction has been made to the Abstract in the Results subsection. The corrected text is as follows:

“Two of these studies consistently demonstrated significant reductions in CFU counts in vitro and changes in oral bacteria in groups treated with D-tagatose alone or in mixtures with other agents compared to controls using other non-caloric sweeteners or placebos (*p* < 0.01).”

There was an error in the original publication [1], regarding missing information related to the PROSPERO registration protocol. A correction has been made to the Materials and Methods section, Design subsection, paragraph one. The corrected text is as follows:

“The protocol adhered to the guidelines of the Preferred Reporting Items for Systematic Reviews and Meta-Analyses (PRISMA); approval code: INPLASY202510002 and approval code: PROSPERO CRD42025632489.”

There was an error in the original publication [1], regarding a typographical error. A correction has been made to the Materials and Methods section, Data Sources and Search subsection, paragraph one. The corrected text is as follows:

“Virtual Health Library (accessed on 31 December 2024).”

There was an error in the original publication [1], regarding a typographical error. A correction has been made to the Results section, paragraph seven. The corrected text is as follows:

“The study by Zakis et al. [13], conducted in the Netherlands, involved 65 adults (ages 18–55 years) and assessed the effect of tagatose on salivary pH and the oral microbiome using 16S rRNA gene amplicon sequencing, red fluorescence of plaque, and a calibrated pH meter.”

There was an error in the original publication [1], regarding missing information. A correction has been made to the Results section, paragraph eleven. The corrected text is as follows:

“In the study by Zakis et al. [13], the participants received mouth rinses D-tagatose, Glucose, Inulin, Isomaltulose and Trehalose at concentrations of 10% for 2 weeks with final observations at the end of 4 weeks.”

There was an error in the original publication [1], regarding a typographical error. A correction has been made to the Results section, paragraph sixteen. The corrected text is as follows:

“The study by Zakis et al. [13] found that mouth rinses with 10% D-tagatose did not significantly change the oral microbiome compared to other sweetener groups. Additionally, no significant changes in salivary pH were observed.”

There was an error in the original publication [1], as the content is incomplete. A correction has been made to the Results section, paragraphs twenty and twenty-one. The corrected text is as follows:

“In the study by Zakis et al. [13], Figures 3–5 offer only a visual summary with limited statistical depth, based solely on *p*-values and frequency percentages, without complementary inferential metrics such as confidence intervals or effect sizes.

Although included in the tables provided, they include different results and do not clarify or complement the data shown in the figures, potentially obscuring the full scope and interpretation of the results.”

There was an error in the original publication [1], regarding a typographical error. A correction has been made to the Discussion section, paragraph eleven. The corrected text is as follows:

“could be limited”.

## Error in Tables

In the original publication [1], there was a mistake in Table 2. In the study by Zakis et al. [13], the country, sample, outcome, and instruments were incorrectly assigned to the corresponding categories. The corrected version of Table 2 appears below.

**Table 2:** The characteristics of the selected studies.

Characteristics	Nagamine et al. [7]	Urrutia-Espinosa et al. [10]	Zakis et al. [13]
Country	Japan	Chile	Netherlands
Sample	19 healthy volunteers (21–49 years)	30 students (18–30 years)	65 healthy volunteers (18–55 years)
Design	RCT	RCT	RCT
Outcome	CFU/mL	CFU/mL Salivary pH	Beta diversity of oral biofilm bacterial taxa Salivary pH.
Instruments	Microbiological crop. Polymerase chain reaction	Microbiological crop. Calibrated pH meter	16S rRNA gene amplicon sequencing, red fluorescence of plaque, and a calibrated pH meter.

RCT: Randomized controlled trial, CFU/mL: colony-forming units/mL. Source: Nagamine et al. [7], Urrutia-Espinosa et al. [10], Zakis et al. [13].

In the original publication [1], there was a mistake in Table 3. In the study by Zakis et al. [13], the intervention of the groups is incomplete. The corrected version of Table 3 appears below.

**Table 3:** The characteristics of the intervention programs.

Characteristics	Nagamine et al. [7]	Urrutia-Espinosa et al. [10]	Zakis et al. [13]
Intervention	EG1: chewing gum with xylitol (5%). EG2: chewing gum with D-tagatose (5%). EG3: D-tagatose gum (2.5%) + 2.5% xylitol (2.5%). CG: placebo.	EG1: D-tagatose mouthwashes (6.4%). EG2: stevia mouthwashes (6.4%). CG: sucrose mouthwashes (6.4%).	EG1: D-tagatose mouthwashes (10%). EG2: Glucose mouthwashes (10%). EG3: Inulin mouthwashes (10%). EG4: Isomaltulose mouthwashes (10%). EG5: Trehalose mouthwashes (10%).
Duration of Study	4 wk	48 h	4 wk

EG: experimental group, CG: control group. Source: Nagamine et al. [7], Urrutia-Espinosa et al. [10], Zakis et al. [13].

In the original publication [1], there was a mistake in Table 4. In the study by Zakis et al. [13], the results were incorrectly assigned to the corresponding categories. The corrected version of Table 4 appears below.

**Table 4:** Comparison of effects of D-tagatose on CFU/mL across studies.

	Nagamine et al. [7]	Urrutia-Espinosa et al. [10]	Zakis et al. [13]
Results	D-tagatose + xylitol significantly decreased CFU/mL compared to control group (*p* < 0.01).	D-tagatose significantly decreased CFU/mL in comparison to sucrose (*p* < 0.001). D-Tagatose did not show significant changes in CFU/mL compared to stevia (*p* = 0.137).	D-tagatose shows no difference significant on salivary pH or microbiome composition compared to baseline.

CFU/mL: colony-forming units/mL. Source: Nagamine et al. [7], Urrutia-Espinosa et al. [10], Zakis et al. [13].

The authors state that the scientific conclusions are unaffected. This correction was approved by the Academic Editor. The original publication has also been updated.
