# Supplementary material for: Effects of D-Tagatose on Cariogenic Risk: A Systematic Review of Randomized Clinical Trials
Source: Nutrients. 2025 Jan 15;17(2):293. doi: 10.3390/nu17020293 (PMC11767683; doi:10.3390/nu17020293)
Supplement: Supplementary file 1 [file nutrients-17-00293-s001.zip › Figure S1. Meta-analysis of the included studies.pdf]

Figure S1. Meta-analysis of the included studies.

OUTCOME: CFU 30 min

D-tagatose vs. Stevia

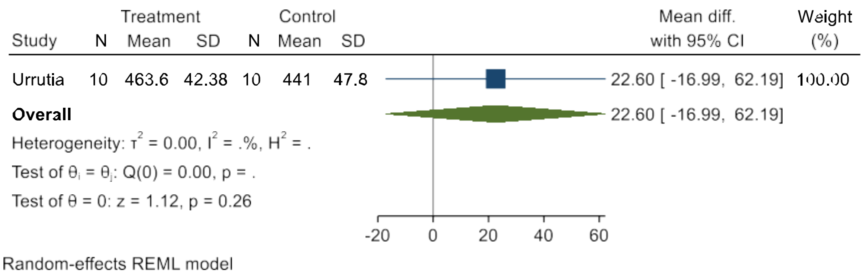

D-tagatose vs. Sucrose

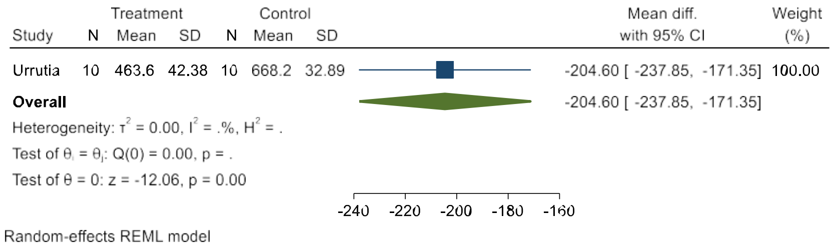

OUTCOME: pH 30 min

D-tagatose vs. Stevia

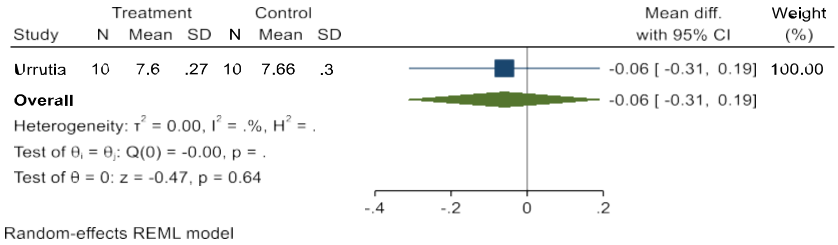

D-tagatose vs. Sucrose

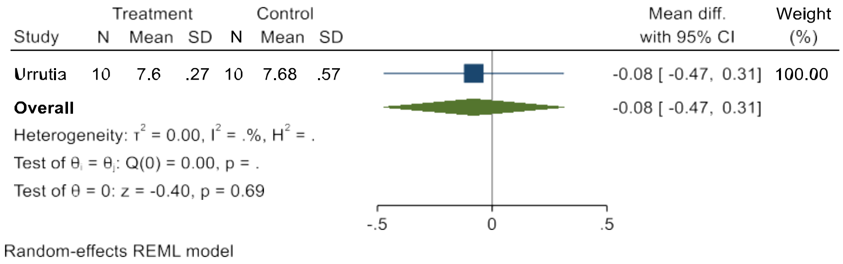

OUTCOME: pH 48 hrs

D-tagatose vs. Sucrose

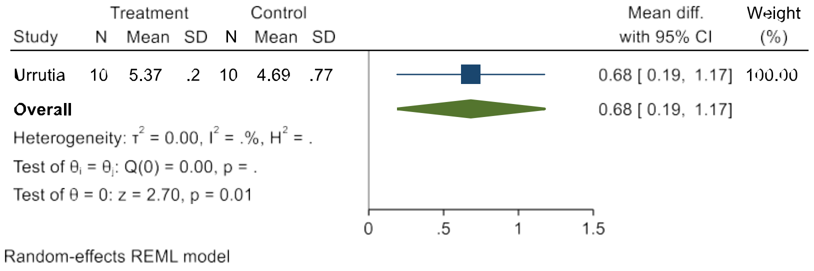

D-tagatose vs. Stevia

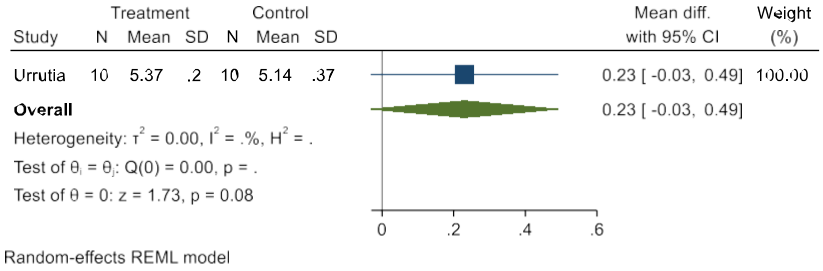

10. Urrutia-Espinosa M, Concha-Fuentealba F, Fuentes-Barría H, Angarita Dávila LC, Carrasco Hernández ME, Aguilera-Eguía R, Alarcón Rivera M, López Soto OP. Effects of D-tagatose, Stevia and Sucrose on pH and oral bacterial activity in dentistry students. A randomized controlled trial. ***Nutr Hosp***. *2024*, 41, 1091-1097.
