# Supplementary material for: Effects of D-Tagatose on Cariogenic Risk: A Systematic Review of Randomized Clinical Trials
Source: Nutrients. 2025 Jan 15;17(2):293. doi: 10.3390/nu17020293 (PMC11767683; doi:10.3390/nu17020293)
Supplement: Supplementary file 1 [file nutrients-17-00293-s001.zip › Table S1. Summary of search strategies.pdf]

Table S1. Summary search strategies

Search strategy for the Medline/Pubmed database.

Number	Search terms
#1	"Tagatose "[Mesh]
#2	"D-tagatose"
#3	or/#1-2
#4	"Dental Caries" [Mesh]
#5	and/#3
#6	Filter: (type_of_study:("Randomized Controlled Trial"))

Search strategy for the Europe PMC

Number	Search terms
#1	"Tagatose "[Mesh]
#2	"D-tagatose"
#3	or/#1-2
#4	"Dental Caries" [Mesh]
#5	and/#3
#6	Filter: (type:("Research articles"))

Search strategy for the CENTRAL

Number	Search terms
#1	"Tagatose "[Mesh]
#2	"D-tagatose"
#3	or/#1-2
#4	"Dental Caries" [Mesh]
#5	and/#3

Search strategy for Scopus

Number	Search terms
#1	"Tagatose "[Mesh]
#2	"D-tagatose"
#3	or/#1-2
#4	"Dental Caries" [Mesh]
#5	and/#3
#6	Filter: (type:("Articles"))

Search strategy for Virtual Health Library

Number	Search terms
#1	"Tagatose "[Mesh]
#2	"D-tagatose"
#3	or/#1-2
#4	"Dental Caries" [Mesh]
#5	and/#3

Search strategy for the Web of Science

Number	Search terms
#1	"Tagatose "[Mesh]
#2	"D-tagatose"
#3	or/#1-2
#4	"Dental Caries" [Mesh]
#5	and/#3
#6	Filter: (type:("Articles"))

Search strategy for the Dentistry & Oral Sciences Source

Number	Search terms
#1	"Tagatose "[Mesh]
#2	"D-tagatose"
#3	or/#1-2
#4	"Dental Caries" [Mesh]
#5	and/#3

Search strategy for the Springer Nature Link

Number	Search terms
#1	"Tagatose "[Mesh]
#2	"D-tagatose"
#3	or/#1-2
#4	"Dental Caries" [Mesh]
#5	and/#3
#6	Filter: (type:("Research Articles"))

Search strategy for BioRxiv

Number	Search terms
#1	"Tagatose "[Mesh]
#2	"D-tagatose"
#3	or/#1-2
#4	"Dental Caries" [Mesh]
#5	and/#3

Search strategy for prepints.org

Number	Search terms
#1	"Tagatose "[Mesh]
#2	"D-tagatose"
#3	or/#1-2
#4	"Dental Caries" [Mesh]
#5	and/#3
#6	and #1-5 [Title/abstract/ Keywords]
