# Supplementary material for: Effects of D-Tagatose on Cariogenic Risk: A Systematic Review of Randomized Clinical Trials
Source: Nutrients. 2025 Jan 15;17(2):293. doi: 10.3390/nu17020293 (PMC11767683; doi:10.3390/nu17020293)
Supplement: Supplementary file 1 [file nutrients-17-00293-s001.zip › Table S2. Summary of studies evaluated in full text excluded.pdf]

**Table S2. Summary of studies evaluated in full text excluded**

| Characteristics | Mayumi et al., [8] | Hasibul et al., [19] | Sawada et al., [20] |
|---|---|---|---|
| Country | Japan. | Japan. | Japan |
| Sample | 18 healthy volunteers. | Streptococcus mutans GS-5. | Streptococcus mutans GS-5. |
| Intervention | D- tagatose (0.1%, 0.5%, 1%, 5%, y 10%). | D- tagatose (1% y 4%). | D- Tagatose (10%). |
| Comparation | Glucose and sucrose | Sucrose and xylitol. | Xylitol, D-psicose, L-psicose, and L-tagatose. |
| Outcome | CFU/mL. | CFU/mL. | CFU/mL. |
| Desings | Descriptive observational | In vitro | In vitro |
| Duration | 48 h | 72 h | 12 h |


8. Mayumi S, Kuboniwa M, Sakanaka A, Hashino E, Ishikawa A, Ijima Y, Amano A. Potential of Prebiotic D - Tagatose for Pre-vention of Oral Disease. *Front Cell Infect Microbiol.* **2021**, *11*, 767944.

19. Hasibul, K.; Nakayama-Imaohji, H.; Hashimoto, M.; Yamasaki, H.; Ogawa, T.; Waki, J.; Tada, A.; Yoneda, S.; Tokuda, M.; Miyake, M.; et al. D-Tagatose Inhibits the Growth and Biofilm Formation of Streptococcus Mutans. *Mol. Med. Rep.* **2018**, *17*, 843–851

20. Sawada D, Ogawa T, Miyake M, Hasui Y, Yamaguchi F, Izumori K, et al. Potent inhibitory effects of D - tagatose on the acid production and water-insoluble glucan synthesis of Streptococcus mutans GS5 in the presence of sucrose. *Acta Med Okayama.* **2015**, *69*, 105-11.
