# Supplementary material for: Effects of D-Tagatose on Cariogenic Risk: A Systematic Review of Randomized Clinical Trials
Source: Nutrients. 2025 Jan 15;17(2):293. doi: 10.3390/nu17020293 (PMC11767683; doi:10.3390/nu17020293)
Supplement: Supplementary file 1 [file nutrients-17-00293-s001.zip › Table S3. Summary of risk of bias.pdf]

Table S3. Summary risk bias.

	Nagamine et al. [7]	Urrutia-Espinosa et al. [10]	Zakis et al. [13]
D1	**Some concerns:** Although a double-blind design is mentioned, it does not detail how the random sequence was generated or assigned, introducing uncertainty.	**Low risk**: An online random sequence generator was used with stratified allocation by center, ensuring proper randomization.	**Some concerns:** Stratified block randomization is described, but it does not specify how the sequence was generated or whether it was fully random.
D2	**Low risk:** Double-blind design executed correctly, with no evidence of significant deviations in the applied interventions.	**Some concerns**: Simple masking with potential staff influence, but no clear evidence of deviations.	**Low risk:** Double-blind design with rigorous monitoring of compliance through the return of used vials by participants.
D3	**Some concerns:** Measurements were taken by a single investigator, but it is not confirmed whether they were blinded, leaving room for potential bias.	**Some concerns:** Although calibrated tools were used to measure pH and CFU, it is not mentioned whether the evaluators were blinded, which could introduce bias.	**Low risk:** Automated methods like genomic sequencing minimize subjective influence in measuring outcomes.
D4	**Low risk:** Although two participants were excluded (due to illness or medications), these exclusions were justified and do not compromise the overall results.	**Low risk:** No participant losses; data from all subjects were included in the analysis, ensuring data integrity.	**Low risk:** Low dropout rate (6%) and proper handling of missing data through intention-to-treat analysis.
**D5**	**Low risk:** Full reporting of all predefined outcomes with appropriate statistical analyses and no evidence of selective reporting.	**Low risk:** All predefined outcomes were reported in line with the study objectives and following CONSORT guidelines.	**Some concerns:** Although key results were reported, it is not detailed whether all predefined outcomes were included or if there was selective reporting.
Overall	**Some concerns:** Insufficient details on randomization and blinding in measurement.	**Some concerns:** Concerns regarding randomization and outcome measurement.	**Some concerns:** Although there is strength in some aspects, uncertainties persist in randomization and selective reporting.

7. Nagamine Y, Hasibul K, Ogawa T, Tada A, Kamitori K, Hossain A, Yamaguchi F, Tokuda M, Kuwahara T, Miyake M. D-Tagatose Effectively Reduces the Number of Streptococcus mutans and Oral Bacteria in Healthy Adult Subjects: A Chewing Gum Pilot Study and Randomized Clinical Trial. Acta Med Okayama. 2020, 74, 307-317.

10. Urrutia-Espinosa M, Concha-Fuentealba F, Fuentes-Barría H, Angarita Dávila LC, Carrasco Hernández ME, Aguilera-Eguía R, Alarcón Rivera M, López Soto OP. Effects of D-tagatose, Stevia and Sucrose on pH and oral bacterial activity in dentistry students. A randomized controlled trial. Nutr Hosp. 2024, 41, 1091-1097.

13. Zakis DR, Brandt BW, van der Waal SV, Keijser BJF, Crielaard W, van der Plas DWK, Volgenant CMC, Zaura E. The effect of different sweeteners on the oral microbiome: a randomized clinical exploratory pilot study. J Oral Microbiol. 2024, 16, 2369350.
